# Comparison of the Effects of Different Negative Pressure Wound Therapy Modes—Continuous, Noncontinuous, and With Instillation—on Porcine Excisional Wounds

**Published:** 2013-10-01

**Authors:** M. Christian Lessing, Roberta B. James, Shannon C. Ingram

**Affiliations:** ^a^Innovation and Strategic Marketing; ^b^Biometrics Data Management, Kinetic Concepts, Inc, San Antonio, Tex

**Keywords:** dynamic NPWT, negative pressure wound therapy with instillation, preclinical model, variable NPWT, wound cleansing

## Abstract

**Objective:** Negative pressure wound therapy (NPWT) can be delivered in continuous or noncontinuous modes, while NPWT with instillation (NPWTi) couples NPWT with automated delivery and removal of topical wound treatment solutions and suspensions. This porcine study compared granulation response of NPWTi (instillation foam dressing with saline) to NPWT (standard foam dressing) in continuous and noncontinuous modes. **Methods:** Full-thickness dorsal excisional wounds in pigs were treated with continuous NPWT, intermittent NPWT, dynamic (controlled variable) NPWT, and NPWTi with saline (n = 10 per group). Wound dimensions were determined from 3D images collected on days 0, 2, 5, and 7. On day 7, animals were euthanized and specimens were harvested for histopathological review. **Results:** Average granulation thickness was not statistically different among continuous (3.29 ± 0.33 mm), intermittent (3.03 ± 0.47 mm), and dynamic (3.40 ± 0.34 mm) NPWT wounds at day 7. Average granulation thickness of NPWTi wounds (4.75 ± 0.54 mm), however, was statistically greater (*P* < .05) by 44%, 57%, and 40%, respectively, than that of wounds treated with continuous, intermittent, and dynamic NPWT. Analysis of 3D images revealed a greater reduction in wound area and perimeter in NPWTi wounds compared to all NPWT wounds (*P* < .05). In addition, the average wound fill rate for NPWTi wounds was faster than that for continuous (40%; *P* < .05), intermittent (25%; *P* > .05), and dynamic (65%; *P* < .05) NPWT wounds. **Conclusions:** Although not confirmed in humans, these porcine data suggest that NPWTi with saline may stimulate a faster rate of wound granulation than NPWT in continuous and noncontinuous modes.

Negative pressure wound therapy (NPWT) creates an environment that promotes wound healing by preparing the wound bed for closure, reducing edema, promoting granulation tissue formation and perfusion, and by removing exudate and infectious material.^1^ These mechanisms and other effects of NPWT have been evaluated in various experimental and clinical studies, ranging from computer models,^2^ to in vitro^3^^,^^4^ and in vivo^5^^-^7 models, to randomized controlled clinical trials.^8^^,^^9^ Depending on the clinician's preference, NPWT can be delivered as continuous pressure or noncontinuous pressure. Two types of noncontinuous NPWT currently exist: intermittent NPWT, in which negative pressure alternates between a set pressure and no pressure for programmed periods of time; and dynamic (variable) NPWT, in which negative pressure transitions between a high pressure and a low pressure following programmed rise and fall times.

In addition, negative pressure wound therapy with instillation (NPWTi) is indicated for patients who would benefit from NPWT as well as controlled delivery and vacuum assisted drainage of topical wound treatment solutions and suspensions, including wound cleansers, over the wound bed.^10^^,^^11^ NPWTi cycles between 3 discrete phases in the following order:

- Instillation—The topical wound treatment solution or suspension is delivered to the wound bed.
- Soak—The topical wound treatment solution or suspension is held in the wound bed for a prescribed period of time.
- NPWT—The topical wound solution, treatment solution or suspension, wound exudates, and infectious materials are removed from the wound bed as NPWT is delivered for a user-selected interval; when the NPWT phase is complete, the cycle begins again with instillation.

Compared to NPWT, NPWTi is a more recent addition to the wound care toolkit and its mechanisms of action are less understood. However, several publications suggest that NPWTi with the appropriate topical wound cleansing solution may help with wound bioburden management.^10^^-^13 Published preclinical studies suggest that noncontaminated wounds may benefit from NPWTi as well.^14^^,^^15^ Lessing et al^14^ showed that porcine excisional wounds treated with NPWTi with saline had 43% more granulation than the wounds treated with continuous NPWT after 7 days of therapy.

Negative pressure is, by design, interrupted during the instillation and soak phases of NPWTi to prevent immediate aspiration of the topical wound treatment solution or suspension. Bench studies suggest that interruption of negative pressure and the introduction of a soak phase is essential for uniform coverage of the wound with the topical wound treatment solution, especially when complex wound geometries are present; if the negative pressure is not stopped while the solution is being delivered, coverage of the wound with the instilled solution may be incomplete.^16^ Thus, this difference between instillation (fluid delivery with a soak phase) and irrigation (fluid delivery without a soak phase) is critical if the topical wound treatment solution requires a prescribed contact time in the wound bed to have a desired effect (eg, to loosen soluble debris, for antisepsis, etc).

Both researchers and device manufacturers claim that all NPWT systems are not created the same, and several studies have suggested that noncontinuous NPWT (intermittent or variable pressure modes) may result in more granulation tissue than continuous NPWT.^5^^,^^17^^,^^18^ However, a separate study in diabetic mice suggested that the granulation response to continuous NPWT is superior to noncontinuous (intermittent and dynamic) NPWT modes.^7^ As such, it is unclear whether the increased granulation tissue observed by Lessing et al^14^ was due to the enhanced wound cleansing provided by NPWTi or the noncontinuous nature of the interrupted negative pressure that is characteristic of NPWTi.

The present study uses a well-controlled porcine model to compare the granulation response of wounds treated with NPWTi (V.A.C. VeraFlo Therapy; KCI USA, Inc, San Antonio, Texas) to those treated with continuous and noncontinuous (intermittent and dynamic) NPWT.

## METHODS

**Figure 1:**
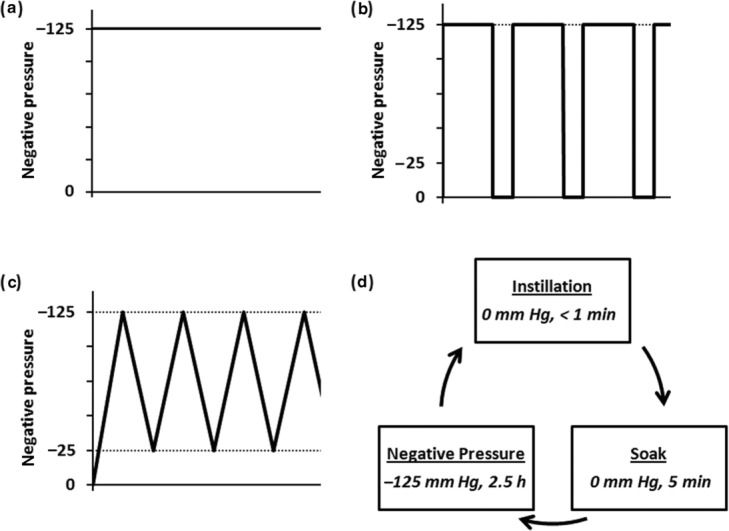
Schematics of negative pressure profiles evaluated in this study: (*a*) continuous NPWT at −125 mm Hg; (*b*) intermittent NPWT with cycles of 5 minutes at −125 mm Hg followed by 2 minutes of 0 mm Hg; (*c*) dynamic NPWT with a 3-minute rise to −125 mm Hg followed by a 3-minute fall to −25 mm Hg; and (*d*) NPWTi with each cycle consisting of a short instillation phase, following by a 5-minute soak phase, followed by a 2.5-h negative pressure phase.

All animal procedures were performed under a protocol approved by the Institutional Animal Care and Use Committee at the test facility. Five female domestic swine (weight range, 65-75 kg) were fully anesthetized and physiological parameters including heart rate, blood pressure, body temperature, and respiratory rate were monitored during the surgical procedure and postsurgical recovery. Each animal received 10 paraspinal (5 per side) dorsal full-thickness 5-cm diameter excisional wounds to the muscle fascia, with the epidermal, dermal, subdermal fat, and subcutaneous fat layers removed. Light pressure with saline moistened gauze was applied to the wound to stop bleeding post excision, as needed. Adjacent or contralateral wounds, as appropriate, were dressed and bridged together as pairs. Bridged wound pairs were assigned to one of the following treatment groups (Fig 1), with each pig having one of every treatment group:

### Continuous NPWT

Following the manufacturer's instructions for use, reticulated open-cell foam (ROCF) dressings (ROCF-G; V.A.C. GranuFoam Dressing; KCI USA, Inc, San Antonio, Texas) were applied to the wounds, bridged together as a pair by overlaying additional ROCF-G, and then covered with drape (V.A.C. Drape; KCI USA, Inc, San Antonio, Texas). The bridged wound pair was connected to a therapy unit (V.A.C.ULTA Therapy System, KCI USA, Inc, San Antonio, Texas) set to deliver continuous NPWT at −125 mm Hg.

### Intermittent NPWT

Wound pairs were dressed as mentioned earlier. The bridged wound pair was connected to a therapy unit (InfoV.A.C. Therapy System, KCI USA, Inc, San Antonio, Texas; Note: the V.A.C.ULTA Therapy System does not provide intermittent NPWT as an option) set to deliver intermittent NPWT, with each cycle consisting of 5 minutes at −125 mm Hg followed by 2 minutes at 0 mm Hg.

### Dynamic (controlled variable) NPWT

Wound pairs were dressed as mentioned earlier. The bridged wound pair was connected to a therapy unit (V.A.C.ULTA Therapy System) that delivered dynamic NPWT, with each cycle consisting of a controlled 3-minute rise to −125 mm Hg followed by a controlled 3-minute fall to −25 mm Hg.

### NPWTi

Following the manufacturer's instructions for use, wounds were dressed with ROCF-V (V.A.C. VeraFlo Dressing; KCI USA, Inc, San Antonio, Texas), bridged together as a pair by overlaying additional ROCF-V, and then covered with drape. The bridged wound pair was connected to a therapy unit (V.A.C.ULTA Therapy System) set to deliver NPWTi with each cycle consisting of instillation of 55 mL of sterile normal saline (instillation phase), a 5-minute soak of saline in the wound (soak phase) and 2.5 hours of negative pressure at −125 mm Hg (NPWT phase). Cycling among these 3 phases continued for the duration of the study period.

A fifth negative pressure treatment mode not commercially available was tested but is not reported here. In total, each treatment group was assigned n = 10 wounds. Dressings were changed on days 2 and 5. Therapy systems were connected to a monitoring system, and alarms were addressed by on-call staff to minimize therapy interruptions. Animals were euthanized on day 7.

Wounds were photographed on Days 0, 2, 5, and 7, and three-dimensional reconstructions of the wounds were generated (3D LifeViz System; QuantifiCare S.A., Sophia Antipolis, France). Wound perimeter and surface area were measured on reconstructions from days 0, 2, 5, and 7. The change in wound volume (percent fill normalized to day 0 wound volume) from day 5 to day 7 was calculated to determine the daily rate of granulation tissue formation during the granulation phase of healing.

After euthanasia, wound tissue was excised en bloc to include underlying musculature and surrounding unwounded tissue. Tissues were fixed in 10% neutral buffered formalin, paraffin embedded, thinly sectioned, and stained with hematoxylin and eosin. Histological sections were evaluated by a board-certified histopathologist. Granulation tissue thickness was measured from the base of the wound to the top of the granulation layer, at 2-mm increments across the entire cross section of the wound; the incremental measurements were averaged together to determine the average granulation thickness for each wound.

Univariate analyses were performed for all data (wound area and perimeter, wound fill, and granulation thickness) and treatment group means with standard error of the mean are presented. Hierarchical, or nested, models were used to compare the treatment group means by including a random intercept to adjust for the potential of animal variation in wound healing. All inferential statistical analyses were performed using a 2-tailed test at α = .05 significance; no adjustments for multiple comparisons were made. All statistical analyses were performed using Statistical Analysis System software (Version 9.3; SAS Institute Inc, Cary, North Carolina).

## RESULTS

### Wound images

**Figure 2:**
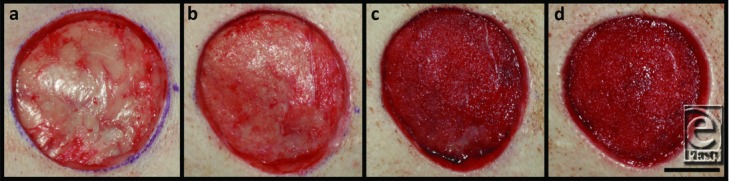
Progression of a single representative wound at (*a*) day 0 creation, (*b*) day 2 dressing change, (*c*) day 5 dressing change, and (*d*) day 7 termination. Note the paucity of granulation tissue at day 2, suggesting the wound has not fully left the inflammatory phase of healing. However, the wound enters the granulation phase of healing by day 5, and a robust granulation layer is present by day 7. (Wound shown was treated with continuous NPWT, scale bar = 2 cm.)

A representative time course of images of a continuous NPWT-treated wound is shown in Figure 2. The 5-mm full-thickness excisional wound immediately following creation has clearly defined edges with the smooth fascia of the underlying muscle intact. At the first dressing change (after 2 days of therapy), the wound appears more pink and with some texture present, but no granulation tissue has formed. By day 5 and continuing to day 7, the wound bed is covered with beefy red granulation tissue.

### Histomorphometry and histopathology

**Figure 3:**
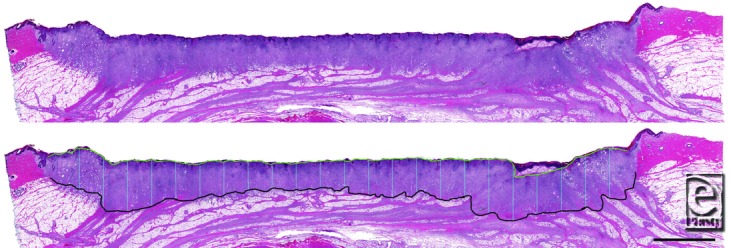
Representative photomicrograph of a wound treated with continuous NPWT. The top image shows the histological section stained with hematoxylin and eosin. The bottom image shows the same section with the bottom (black tracing) and top (green tracing) of the granulation tissue marked. The vertical blue lines are the incremental granulation tissue thickness measurements, spaced every 2 mm. These increments are averaged to determine the average granulation tissue thickness for this wound. (Scale bar = 5 mm)

**Figure 4:**
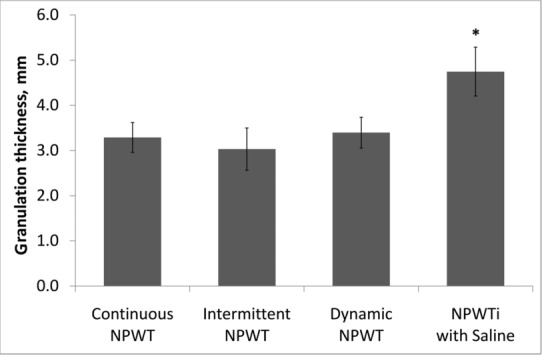
Average granulation tissue thickness measured in histology specimens at day 7. Data are shown as mean ± standard error of the mean. (n = 10 wounds per group; *P < .05 for NPWTi compared to all NPWT groups)

A representative photomicrograph of a continuous NPWT-treated wound is shown in Figure 3, with incremental granulation tissue thickness measurements indicated. The average granulation tissue thickness at day 7 for each treatment group is shown in Figure 4. There was no statistical difference between the 3 NPWT treatment groups (continuous NPWT [3.29 ± 0.33 mm], intermittent NPWT [3.03 ± 0.47 mm], or dynamic NPWT [3.40 ± 0.34 mm]). However, the NPWTi with saline treatment group (4.75 ± 0.54 mm) was significantly greater than all of these (*P* < .05). The granulation tissue in the NPWTi with saline treatment group was 44% thicker than in continuous NPWT, 57% thicker than in intermittent NPWT, and 40% thicker than in dynamic NPWT.

### Three-dimensional wound reconstruction measurements

**Figure 5:**
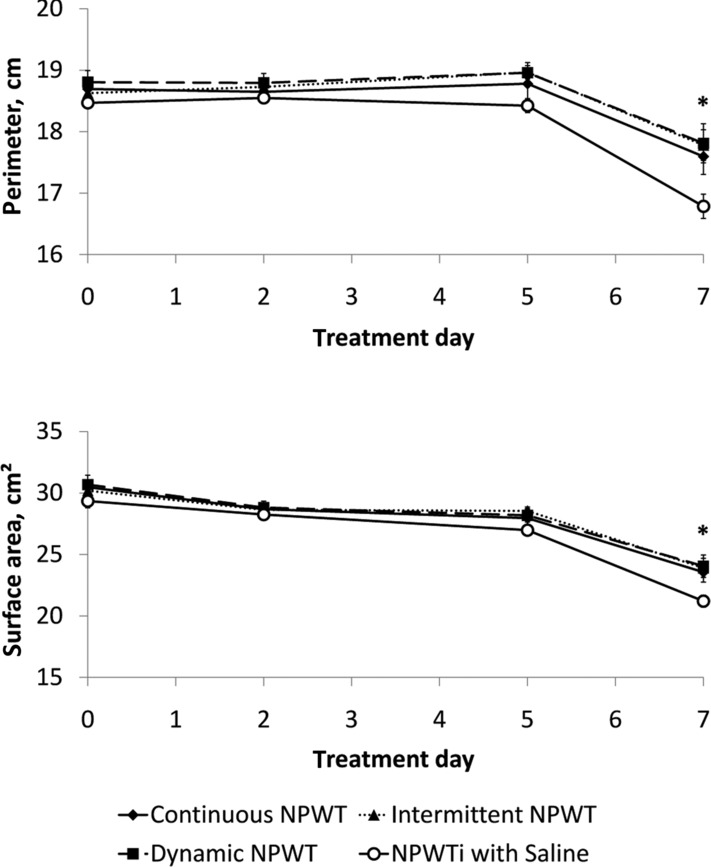
Changes in wound perimeter (*top*) and surface area (*bottom*) calculated from 3D wound reconstructions. Data are shown as mean ± standard error of the mean. (n = 10 wounds per group, *P < .05 for NPWTi compared to all NPWT groups.)

Changes in wound perimeter and wound surface area from day 0 to day 7 are shown in Figure 5. These figures show a decrease in wound perimeter and wound surface area with statistically significant smaller mean values at day 7 for wounds treated with NPWTi with saline compared to wounds treated with continuous, intermittent, or dynamic NPWT modalities (*P* < .05).

**Figure 6:**
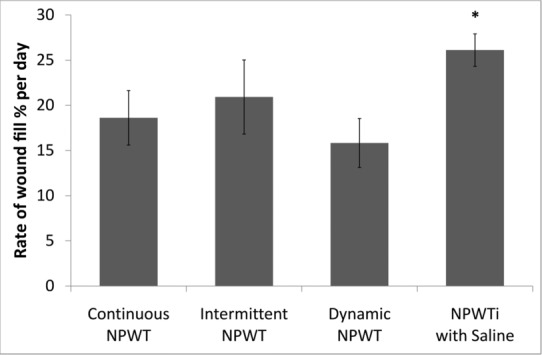
The rate of change of wound volume, calculated as percent fill per day. Data are shown as mean ± standard error of the mean. (n = 10 wounds per group, *P < .05 for NPWTi compared to continuous NPWT and dynamic NPWT.)

The rates of wound volume (percent fill per day) from day 5 to day 7 were: continuous NPWT, 18.6% ± 3.0% per day; intermittent NPWT, 20.9% ± 4.1% per day; dynamic NPWT, 15.8% ± 2.7% per day; and NPWTi with saline, 26.1% ± 1.8% per day (Fig 6). The rate of wound fill with NPWTi with saline was statistically faster (*P* < .05) than with continuous NPWT (40% faster) or dynamic NPWT (65% faster); however, the difference in wound fill rate between NPWTi with saline and intermittent NPWT treatment group was not statistically significant (25%, *P* > .05).

## DISCUSSION

The first documented uses of NPWTi have suggested the therapy to be important in the management of contaminated wounds with measurable bioburden.^13^^,^^19^ However, several preclinical studies have suggested that the cleansing provided by NPWTi may also benefit noncontaminated wounds. In 2010, Leung et al published a study on pigs showing that NPWTi with saline elicited a faster rate of wound filling with granulation and increased collagen content compared to continuous NPWT.^15^ In subsequent work by Lessing et al,^14^ the differences in granulation were more pronounced: the granulation tissue in porcine wounds treated with NPWTi with saline was 43% thicker than in wounds treated with continuous NPWT after 7 days of therapy.^14^ However, as mentioned previously, these studies were interpreted with caution, because it was unknown whether the increase in granulation tissue was due to wound washing and cleansing or due to the noncontinuous nature of NPWTi.

This study compares, for the first time, continuous and noncontinuous (intermittent and dynamic) NPWT profiles to NPWTi. Despite prior reports by Morykwas et al^5^ and Malmsjo et al^17^ associating noncontinuous negative pressure with more granulation tissue than continuous negative pressure, no significant differences were observed between the continuous and noncontinuous NPWT groups for all evaluated outcomes in this study.

A limitation of the NPWT systems used in the studies of Morykwas et al^5^ and Malmsjo et al^17^ could explain this difference: less sophisticated NPWT systems measure the pressure at or near the pump, but not at the wound bed. Pressure measurement at the wound is critical to the pump's adjustment to challenges that develop in the tubing during the course of therapy, such as changes in exudate volume and viscosity, the vertical distance between the pump and the wound (referred to as *head height*), leaks, and blockages. Studies have shown that, when faced with these challenges, negative pressure systems or other vacuum sources that did not measure the pressure at the wound failed to deliver the set negative pressure level to the wound bed.^20^^,^^21^ As stated by Ahearn^18^ in 2009 review, “suboptimal strain in wounds may result in suboptimal healing.” The therapy units used in the present study measured pressure at the wound bed, and review of the therapy and alarm logs indicate that there were no persistent therapy interruptions or deviations.

Continuous NPWT and NPWTi have been shown to create an environment that supports wound healing by promoting granulation tissue formation, so a subset of the analyses were designed to determine the rate of new tissue formation during this granulation phase. The canonical understanding of skin wound healing states that healing occurs in discrete, but overlapping, phases following injury: hemostasis (complete in the first minutes to hours postinjury), inflammation (beginning at injury but continuing for several days), proliferation and granulation (beginning 3-5 days postinjury), and remodeling (beginning several weeks postinjury).^22^ Thus, the day 0 and day 2 time points were excluded from the evaluation of the rate of granulation tissue formation. No statistically significant differences in the rate of change of wound volume (percent fill increase) were observed between day 5 and day 7 in the continuous and noncontinuous NPWT groups (Fig 6); however, NPWTi-treated wounds exhibited a faster increase in wound volume fill compared to the continuous and noncontinuous NPWT groups. Furthermore, wounds treated with NPWTi showed larger reductions in wound perimeter and surface area than with either continuous or noncontinuous NPWT, suggesting faster wound size reductions in NPWTi-treated wounds.

These data support the hypothesis that the mechanism for the increased granulation response with NPWTi is extended, deliberate wound cleansing beyond the initial debridement rather than the intermittent nature of the therapy. The layer of exudate on a wound has evolved to serve as a barrier to infection, and its components play important roles in wound healing: for example, cytokines attract host cells including immune and inflammatory cells, reactive oxygen species target microorganisms, and proteolytic enzymes including matrix-metalloproteinases break down devitalized tissue. However, when uncontrolled, these exudate components contribute to wound chronicity. Consequently, exudate management is a pillar of modern wound bed preparation, along with debridement and bioburden management.^23^ NPWTi dilutes and removes excess wound exudates and infectious materials, and it can be considered as an extension of the initial wound debridement and cleansing. A clean wound environment may allow the limited cellular metabolic and energetic resources in the wound to be dedicated to healing pathways, including cell proliferation and matrix production, as opposed to immune and inflammatory responses.

After NPWTi is prescribed, a topical wound treatment solution must be selected and a protocol developed. Considerations for selection of a solution should include: wound type and overall status, known or suspected bioburden (level and type), goals of therapy, patient allergies, and recommendations of the solution manufacturer related to solution soak time and application frequency. Given the complexity and number of permutations of these factors, each wound should be evaluated individually and its NPWTi treatment program developed and revised as the wound progresses.

Compatibility of the solution with the NPWTi system and its components (including the tubing, dressing, drape, and adhesive) should also be considered, as reactive topical wound solutions could have adverse interactions with the components. A list of compatible solutions can be obtained from the manufacturer.

Recent clinical publications report the use of NPWTi-compatible topical wound treatment solutions, including the following.

- Polyhexanide on osteomyelitis of the pelvis or lower extremity,^24^ skin and soft tissue wounds,^25^ chronic or acute orthopedic wounds,^12^ and infected and surgically debrided wounds^26^
- Saline or sterile water on complex wounds and wounds where conventional NPWT was ineffective^27^
- Hypochlorite-based solutions on venous stasis ulcers^28^ and various other wounds^11^
- Silver nitrate on complex wounds.^29^

Other NPWTi-compatible solutions include mafenide acetate, octenidine dihydrochloride, acetic acid (ethanoic acid), and benzalkonium chloride; anecdotal reports of their use on individual patients are not yet available in the peer-reviewed literature. It is unknown how solutions other than saline may affect granulation tissue formation clinically.

Noncontinuous pressure modes will continue to play an important role in NPWT. At the subcellular level, the cytoskeleton of many cell types can adapt rather quickly to microdeformational changes.^30^^,^^31^ As such, intermittent NPWT is, anecdotally, used to jump-start stalled wounds that have quit responding to continuous NPWT. However, intermittent NPWT is not commonly used at the beginning of a wound-treatment program because of the potential of patient discomfort associated with the contraction and expansion of dressings that occurs as negative pressure transitions between the therapeutic set point and 0 mm Hg. Dynamic NPWT has been developed to increase patient comfort during noncontinuous NPWT—the rate of pressure change is controlled, and the pressure does not drop below −25 mm Hg, preventing expansion of the dressing. Additional studies are needed to assess the effectiveness of intermittent and dynamic NPWT modes and their role as a clinical option for chronic and stalled wounds.

As with any preclinical evaluation of a medical device, the relevance of the model is open for discussion. The porcine excisional wound model is an acute injury in a young, healthy animal, and these wounds will generally heal without requiring advanced therapies; however, the porcine model is the accepted preclinical model for wound healing.^32^ Even with the limitations of the model, demonstration of a difference between advanced treatments is noteworthy; each treatment group was represented in duplicate in each pig, so internal controls were present. This study would be challenging to repeat in humans, because the heterogeneity of clinical wounds and lack of internal controls would require much larger sample sizes. Also, determination of granulation tissue thickness is only possible as a destructive measurement (ie, tissue must be collected as a biopsy or other excisional method to determine the thickness).

Finally, as dressing choice is often a topic of discussion in the literature, it should also be mentioned here that new ROCF dressings have been specially designed for use with NPWTi.^14^ These dressings have both increased mechanical properties to reduce the likelihood of tearing and fragmentation and reduced hydrophobicity, which improves their ability to distribute fluids within the wound bed.

Together these preclinical data suggest that wounds treated with NPWTi with saline instillation may exhibit faster granulation rates than wounds treated with either continuous or noncontinuous NPWT. The granulation response of NPWT may be tied to many factors, including wound microstrain, increased perfusion, edema reduction, and removal of exudate and debris that may impair wound healing. NPWTi provides these factors as well as automated delivery and removal of topical wound treatment solutions and suspensions with a controlled soak time. The soak phase provided by NPWTi also allows for controlled solution exposure times and may help to loosen soluble debris and exudate while increasing solution coverage of the wound bed, providing a cleaner environment for healing. Ultimately, further investigations to understand mechanisms of action of NPWTi and noncontinuous NPWT are warranted, and the significance of these findings must be confirmed in clinical studies.
